# Effects of eating breakfast on children and adolescents: A systematic review of potentially relevant outcomes in economic evaluations

**DOI:** 10.29219/fnr.v63.1618

**Published:** 2019-09-12

**Authors:** Martina Lundqvist, Nicklas Ennab Vogel, Lars-Åke Levin

**Affiliations:** Department of Medical and Health Sciences, Linkoping University, Linköping, Sweden

**Keywords:** children, adolescents, youth, breakfast, effects, review

## Abstract

**Background:**

Breakfast is often described as the most important meal of the day. Several studies have focused on examining if breakfast habits have any short-term effects on school attendance, academic achievement, and general health in children and adolescents. Informed decisions of whether to promote eating breakfast or not require a more long-term perspective.

**Objective:**

The aim of this study was to conduct a systematic review of scientific publications studying the effects identified as potentially relevant for the economic evaluation of eating breakfast in children and adolescents.

**Design:**

A systematic literature review was conducted. Studies were identified by searching the electronic databases PubMed, CINAHL, Web of Science, and PsycINFO between January 2000 and October 2017. The inclusion criteria applied were published articles from peer-reviewed journals with full text in English, quantitative studies collecting primary data with school-aged children, and adolescents aged from 6 to 18 years as participants, performed entirely or partly in countries with advanced economies, except Japan and Taiwan.

**Results:**

Twenty-six studies fulfilled the inclusion criteria, and studies that were judged to be of at least moderate quality were included in the analysis. The results of the review of eating breakfast studies showed positive and conclusive effects on cognitive performance, academic achievement, quality of life, well-being and on morbidity risk factors.

**Conclusions:**

The overall assessment of the studies indicated positive effects of eating breakfast. How the identified effects influence societal costs and an individual’s quality-adjusted life years require further research.

## Popular scientific summary

- The article reviews the potential study outcomes for the economic evaluation of long-term effects of eating breakfast in children and adolescents.
- The studies showed positive and conclusive effects on cognitive performance, academic achievement, quality of life, well-being, and on morbidity risk factors.
- How the identified effects influence societal costs and an individual’s quality-adjusted life years require further research.
- Development of simulation models to estimate the long-term costs and effects of eating breakfast should be considered.

Breakfast is often described as the most important meal of the day. Several studies have focused on examining if breakfast habits have any bearing on school attendance, academic achievement, and general health in children and adolescents. Research findings indicate that the regularity of wholesome, daily meal patterns comprised of healthy foods is important for the physical and mental development of children and adolescents (1). The association between adiposity and breakfast habits among children and adolescents is a well-studied topic (2). The quality and regularity of breakfast consumption have also shown to be associated with mental performance, academic achievement, physical activity, and quality of life (3–9). Despite this, young people often skip breakfast (10), and the prevalence of children and adolescents skipping breakfast is increasing (11). In addition, it has been shown that breakfast skipping is particularly common amongst children of lower socioeconomic status (12).

As societal resources are scarce, and needs are endless, choices between health interventions must be made. Several published reviews have examined varying short-term effects of eating breakfast on children and adolescents (6, 13–15). However, informed decisions of whether to promote eating breakfast or not require a more long-term perspective. Economic evaluation seeks to identify, quantify, and compare the long-term costs and effects of different interventions for well-founded and informed decision-making. The costs are weighed against the health effects measured in such a way that it corresponds to a value, usually quality-adjusted life years (QALYs). This measure should ideally encapsulate the impact of an intervention on a person’s length of life and the impact on their health-related quality of life. To our knowledge, it remains unstudied which short-term effects of breakfast eating that may serve as parameters for modeling long-term effects of eating breakfast in economic evaluations. As a starting point for future economic assessments, our intention was to review study outcome measures and identify those that could transform into long-term QALYs.

The aim of this study was to conduct a systematic review of scientific publications studying effects identified as potentially relevant for the economic evaluation of eating breakfast in children and adolescents.

## Methods

### Search methods for identification of studies

Studies were identified by searching the electronic databases PubMed, CINAHL, Web of Science, and PsycINFO between January 2000 and October 2017.

To capture all relevant articles published in the field, two different search strategies were constructed (see Table 1).

**Table 1:** Search strategies

Search strategy 1
*Breakfast* AND *Children OR Adolescents OR Teen OR Youth OR Students OR Kids OR Pupils* AND *Behavior OR Mental OR Learning OR Effect OR Cognitive OR Academic OR Achievement OR Performance OR Well-being OR Quality of life OR Health*

**Search strategy 2**
*School* AND *Breakfast* AND *Children OR Adolescents OR Teen OR Youth OR Students OR Kids OR Pupils* AND *Behavior OR Mental OR Learning OR Effect OR Cognitive OR Academic OR Achievement OR Performance OR Well-being OR Quality of life OR Health*

### Selection of studies

One of the authors (ML) conducted the search in October 2017. Initially, two of the authors (ML and NEV) read all titles and abstracts of the identified studies to determine the relevance of each article. If title and abstract met with the inclusion criteria, the study proceeded to the next stage of the review process. Studies with insufficient information in title and abstract were also proceeded to the next stage of the review process. After this selection, all authors (ML, L-ÅL, and NEV) read the remaining studies in full text, in order to confirm the inclusion eligibility and conduct the quality assessment.

The inclusion criteria applied in the review were determined before the initial search. The criteria are presented as follows:

- Published articles from peer-reviewed journals with full text in English.
- Studies collecting primary data.
- Quantitative studies.
- Study participants in the age of 6 to 18 years.
- Studies performed entirely or partly in countries listed by Central Intelligence Agency (CIA) as advanced economies, except Japan and Taiwan (16).
- Studies using clear definitions of eating and not eating breakfast.

### Review of quality

In pairs, the authors read the articles in full to assess both eligibility and scientific quality. If the two authors who made the initial assessment did not agree, the third author will also read the article. The scientific quality assessment was based on the following criteria: adequate control group(s), randomization, sufficient statistical power, control of confounders, sufficient descriptions of experimental design, relevance of outcome measures, and consistency between reported results and conclusions (17, 18).

During the selection process, the authors assessed the relevance of the outcome measures (i.e. study effects). Study effects deemed to have the potential to be transformed into long-term effects were selected and categorized into four topics: academic achievement, quality of life and well-being, morbidity risk factors, and cognitive performance. Studies focusing on the association between adiposity, obesity, overweight, and eating breakfast were excluded, because the topic has already been studied thoroughly. Studies using other outcome measures were also excluded.

In order not to let the results of the study affect the quality assessment, this was done separately without, as far as possible, reading the study results. The criteria for quality assessment varied with different study types. The quality of the studies was rated as high, moderate, or low. A study rated “high quality” had to fulfill all the quality criteria. Studies of moderate quality fulfilled several of the criteria. Finally, low-quality studies either failed to meet several of the criteria or had major shortcomings in certain criteria. Studies rated as being of high or moderate quality were included in the further analysis. The low-quality studies were excluded. They are presented with a comment in Appendix 1. Table 2 contains the following information from the included studies: reference, participant characteristics (number, age, gender), study design, study purpose, outcomes, authors conclusions, and the scientific quality assessment.

**Table 2:** Summary of studies included in the review

First author (year), country	Participants			Study design	Study purpose	Outcomes	Author conclusion	Scientific quality assessment
	*N*	Age	Gender (female)					
**Morbidity risk factors**								
Hallstrom et al. (2013), SE, ES, BE, DE, FR GR, IT, AU (31)	2,929	14.7 years	53%	Cross-sectional, observational	To examine the association between breakfast consumption and cardiovascular disease (CVD) risk factors in European adolescents.	Cardiorespiratory fitness Physical activity Serum triacylglycerol (TAG) Total cholesterol (TC) High-density lipoprotein-cholesterol (HDL-C) Low-density lipoprotein-cholesterol (LDL-C) Glucose	Findings regarding European adolescents confirm previous data indications: adolescents who consume breakfast regularly have lower body fat content than other peers. Results also show that regular breakfast consumption (BC) is associated with higher cardiorespiratory fitness and (especially in male adolescents) with a healthier cardiovascular profile and negation of the effect of excess adiposity on TC and LDL-C.	Moderate
Marlatt et al. (2016), US (32)	367	14.7 years	49%	Observational	To evaluate the relationship between both breakfast and fast food consumption on selected biomarkers and important cardiovascular and metabolic risk factors among healthy adolescents, and further examine the relationship between these dietary behaviors and the known risk factor clustering that occurs with the metabolic syndrome.	Body mass index (BMI) Percent body fat (PBF) S-, DBP L-, HDL Triglyceride (TG) Glucose Insulin Homeostasis Model Assessment-Insulin Resistance Index (HOMA-IR) metabolic syndrome (MetS) cluster score	The finding suggests that fast food and BC are associated with some metabolically important chronic disease risk factors in healthy adolescents.	Moderate
Moschiano et al. (2012), IT (33)	800	10≤ years	40.6%	Observational	To assess the possible association between headache and specific habits and lifestyle factors.	Headache	Evidence of clear association between headache and irregular intake of meals (especially irregular breakfast) and sleep disturbance with significant differences when comparing subjects with and without headache.	Moderate
Papoutsou et al. (2014), CY, GR, DE, IT, SE, EE, BE, ES (29)	8,863	2 < 10 years	48.8%	Cross-sectional Observational	To investigate the relationship between breakfast routine and CVD risk factors in a multinational sample.	Blood glucose TC LDL-C HDL-C TG Physical activity (PA)	Daily BC contributes to controlling school-aged children's weight and lipid profile. It promotes higher PA.	Moderate
Sese et al. (2012), ES, GB, FR, BE, DE, AU, HU, GR(34)	826	14.8 years	52%	Observational	To examine the associations of food behaviors and preferences with markers of insulin resistance and clustered metabolic risk factors score after controlling for potential confounders, including body fat in European adolescents.	TG TC HDL-C Blood glucose School Breakfast Program (SBP) HOMA-IR	The results of this study indicate that insulin resistance and a clustered metabolic risk factors score are positively associated with food behaviors and preferences. Skipping breakfast explains part of the insulin resistance variance.	Moderate
Smith et al. (2010), AU (30)	2,184	N/A	53.3%	Longitudinal, observational; follow-up period: 21 years.	To examine longitudinal associations of breakfast skipping in childhood and adulthood with cardiometabolic risk factors in adulthood.	Mean weight Circumference Cardiometabolic risk factors	Participants skipping breakfast in both childhood and adulthood had larger waist circumferences, higher BMIs, and poorer cardiometabolic profiles than did those who reported eating breakfast at both time points.	Moderate
Walter (2014), US (35)	13,570	11–17 years	51%	Cross-sectional	To study how lifestyle behaviors (skipping meals, water intake, tobacco use, alcohol use, and physical activity) and illness-related factors (depression, somatic complaints, insomnia, and obesity) work together to predict headache in an adolescent population.	Recurrent headache	Lifestyle behaviors and illness-related factors are associated with adolescent headache. Skipping breakfast three or more times was one of them.	Moderate
Wennberg et al. (2015), SE (36)	889	16 years	52.2%	Longitudinal, observational, follow-up period: 27 years.	To analyze whether poor breakfast habits in adolescence predict the metabolic syndrome and its components in adulthood.	Metabolic syndrome Central obesity High fasting glucose	Poor breakfast habits in adolescence predicted the metabolic syndrome in adulthood. Of the metabolic syndrome components, poor breakfast habits in adolescence predicted central obesity and high fasting glucose in adulthood.	Moderate
Wennberg et al. (2016), SE (37)	889	16 years	52.2%	Longitudinal, observational; follow-up period: 27 years.	To investigate whether irregular eating of meals in adolescence predicts the metabolic syndrome and its components in adulthood, and if any specific meal is of particular importance.	Metabolic syndrome	Irregular eating of meals in adolescence predicted the metabolic syndrome in adulthood, but not independently of BMI and lifestyle in adolescence. Poor breakfast in adolescence was the only specific meal associated with future metabolic syndrome, even after adjustments.	Moderate
**Cognitive performance**		
Cooper et al. (2011), GB (50)	96	13.3 years	62.5%	Randomized crossover design	To examine the effects of breakfast consumption on cognitive function, mood and blood glucose concentration in adolescent schoolchildren.	Modified Activation–Deactivation Checklist (AD ACL) (mood questionnaire) Visual Analogue Scale (VAS)—hunger, fullness Blood glucose concentration cognitive function (CF) tests: Visual search (focused attention) Stroop (negative priming) Sternberg paradigm (working memory)	BC improved the accuracy of responses on the visual search and Stroop tests. BC also improved response times on the more complex levels of the Sternberg paradigm, but did not have consistent effects on response times on the other tests conducted. BC was particularly beneficial for the more cognitively demanding tasks, whereas the simpler tasks could be performed to a similar level following breakfast omission.	Moderate
Defeyter and Russo (2013), GB (49)	40	14.2 years	52.5%	Crossover design	To investigate the effect of breakfast consumption on cognitive performance and mood in adolescents, and any interaction that breakfast consumption might have with cognitive load.	Bond-Lader (mood scale) VAS—thirst, hunger, satiety Cognitive load (CL) tests: Delayed word recall (memory) Choice reaction time (attention) Rapid visual information processing task (RVIP, sustained attention) Stroop (negative priming) Serial 3s, 7s (attention, memory)	Overall, it appeared that after breakfast, participants felt more alert, satiated, and content. Only in the recall task did performance appear to be significantly modulated by the interactive combination of the effect of BC and task difficulty, with improved performance at time two when the task was harder.	Moderate
Hjorth et al. (2016), DK (27)	710–828	9.9 years	49%	Cluster-randomized crossover design	To examine the independent associations between weight status and lifestyle indicators with cognitive performance in 8- to 11-year-old Danish children.	Children’s Sleep Habits Questionnaire (CSHQ) Cardiorespiratory fitness (CRF) Aufmerksamkeits-Belastungs test (d2-test) (concentration) Sentence reading speed and correctness Mathematics proficiency	Normal weight children had higher cognitive performance compared to overweight/obese and underweight children. Daily BC was associated with higher cognitive performance in the d2-test, mathematics and/or sentence-reading test.	Moderate
Wesnes et al. (2003), GB (38)	29	12 years	51.7%	Randomized, four-way crossover design	To determine the extent to which breakfast cereals would help to prevent declines in cognitive function in school children.	Cognitive drug research (CDR) test: Word presentation, immediate word recall, picture presentation, simple reaction time, digit vigilance, choice reaction time, spatial and numeric working memory, delayed word recall, word and picture recognition (attention, working memory, episodic secondary memory) Bond and Lader (mood, alertness)	Skipping breakfast impairs attention and episodic memory, increasing in magnitude over the morning. Ingesting carbohydrates in the form of breakfast cereals reduces attention deficit by more than half and, for some aspects of memory (immediate word recall), prevents the deficit altogether. No benefits to attention or episodic memory with the glucose drink; in fact, greater initial impairment with the drink than with no drink or breakfast. Improvements in alertness and contentment did occur for 90 min following the glucose drink, but effects faded thereafter, whereas the benefits continued from the two cereals.	Moderate
Wesnes et al. (2012), GB (39)	1,386	10.59 years	52%	Controlled trial	To determine the extent to which breakfast cereals would help to prevent declines in cognitive function in school children.	Power of attention Response speed variability Digit vigilance task Choice reaction time task Picture recognition	Power of Attention, a score reflecting the ability to focus attention and avoid distraction, was slowed by 7% in those children who did not have breakfast. The ability to sustain attention was also compromised, 7% less targets being detected in the digit vigilance task while 23% more false alarms were made. The ability to correctly identify pictures was impaired by 9% and speed was slowed by 9%. Finally, the response speed variability was 10% greater in children who did not have breakfast. These scores reflect every aspect of cognitive performance assessed, showing a comprehensive difference between the two groups.	Moderate
Widenhorn-Müller (2008), DE (40)	104	17.2 years	46%	Randomized crossover design	To determine whether breakfast had effects on the cognitive performance and mood of high school students.	d2-Test (concentration speed and attention) Lern- und Gedaechtnistest (LGT-3, learning capacity, immediate memory) Verfahren zur Erfassung des Gefühlszustandes (VGZ, mood assessment scale)	This crossover trial demonstrated positive short-term effects of breakfast on cognitive functioning and self-reported alertness in high school students.	Moderate
**Quality of life and well-being**								
Page et al. (2009), US, SK, HU, RO, CZ (41)	3,121	16.6 years	54.7%	Cross-sectional	To investigate self-rated health (SRH) in Central and Eastern European (CEE) adolescents and determine its association with psychosocial functioning and other dimensions of adolescent health.	Self-Rated Health R-UCLA Loneliness Scale Beck Hopelessness Scale Cheek and Buss Shyness Scale MacArthur Scale of Subjective Social Status – Youth Version Self-Rated Happiness	Self-rated Health appears to be associated with psychosocial functioning and other dimensions of adolescent health in CEE youth. Eating breakfast was one of 12 significant predictors of SRH.	Moderate
Richards and Smith (2016), GB (42)	2,307	13.6 years	51.5%	Longitudinal study with two cross-sections; follow-up period: 6 months.	To investigate the effects of consuming energy drinks and missing breakfast on stress, anxiety, and depression in a cohort of secondary school children.	The Diet and Behavior Scale **(**DABS) Exercise frequency questionnaire Self-Assessed Mental Health (Wellbeing Process Questionnaire [WPQ]-items)	The current study has provided evidence to suggest that high stress, anxiety, and depression levels in adolescents are associated with breakfast omission. The relationship is unlikely to be causal in nature and there may be bi-directional mechanisms involved, with mental health also influencing whether or not breakfast is consumed.	Moderate
Smith (2010), GB (51)	213	8.11 years	50.7%	Separate groups design	To examine the effects of consuming different breakfast cereals on parents' perceptions of the alertness, cognitive function and other aspects of the well-being of their children.	Questionnaire measures of well-being (alertness, cognitive difficulties, anxiety, depression, emotional distress, fatigue, somatic symptoms, positive/negative mood, symptoms, bowel problems).	Breakfast cereal consumption by children is associated with greater well-being.	Moderate
**Academic achievement**		
Boschloo et al. (2012), NL (43)	605	14.81 years	56%	Cross-sectional	To investigate whether adolescents who habitually skip breakfast have lower end-of-term grades than adolescents who eat breakfast daily.	BC Attention Problem Scale School performance—arithmetic mean of subjects Dutch, mathematics and English.	Study shows that breakfast skipping and school performance are related, partially mediated by attention. No causal conclusions drawn.	Moderate
Burrows et al. (2017), AU (44)	4,245	11.33 years	50.55%	Observational	To conduct secondary analysis to examine associations between a range of dietary behaviors and children's academic achievement.	Dietary behaviors National Assessment Program Literacy and Numeracy (NAPLAN) (reading, writing, spelling, grammar/punctuation, numeracy)	The findings demonstrate the association between dietary behaviors and higher academic achievement. Breakfast was only significantly associated with the academic domain of writing.	Moderate
Faught et al. (2017), CA (48)	28,608	14.1 years	50.9%	Observational	To characterize the associations between health behaviors and self-reported academic achievement.	Questionnaire (academic achievement, PA, healthy eating habits, sleep, screen time, body weight [BW]-status, socioeconomic status [SES])	The present findings demonstrate that lifestyle behaviors are associated with academic achievement.	Moderate
Lien (2007), NO (45)	7,305	15–16 years	50.6%	Cross-sectional survey	To examine the relationship between mental distress, academic performance and regular breakfast consumption across gender and immigration status.	Average grade for mathematics, written Norwegian, English and social science. Hopkins Symptoms Checklist (10-Item Version) (HSCL-10) (mental distress)	The implications of skipping breakfast on mental distress and academic performance are stronger for boys than girls and stronger for Norwegians than immigrants.	Moderate
Littlecott et al. (2016), GB (5)	3,093 (baseline), 3,055 (follow-up)	9–11 years	50.8% (baseline) 49.5% (follow-up)	Observational	To examine the link between breakfast consumption in 9- to 11-year-old children and educational outcomes obtained 6–18 months later.	Educational outcomes: scholastic assessment test (SAT)-scores	Significant positive association between self-reported BC and educational outcomes.	Moderate
Ptomey et al. (2016), US (28)	698	7.5 years	50.5%	Cluster-randomized controlled trial	To determine whether breakfast consumption or content affects academic achievement measured by standardized tests.	Wechsler individual achievement test (3-components) (WIAT-III)	Both BC and breakfast content may be associated with improved standardized test performance in elementary school students.	Moderate
Sampasa-Kanyinga & Hamilton (2017), CA (47)	9,912	15.2 years	48.6%	Observational	To investigate the association between breakfast consumption and school connectedness and to extend previous research on the association between breakfast consumption and academic achievement.	School connectedness (questionnaire) Academic performance (good marks: 70%–100%, poor marks: <70%)	Provides supporting evidence for the association between regular BC and higher school connectedness and academic performance.	Moderate
Stea and Torstveit (2014), NO (46)	2,432	15–17 years	51%	Cross-sectional study	To examine the associations between several lifestyle habits and academic achievement in adolescent girls and boys.	Self-reporting questionnaire (dietary, PA, smoking, and snuffing habits, academic achievement)	Regular meal pattern, intake of healthy food items and being physically active were all associated with increased odds of high academic achievement, whereas the intake of unhealthy food and beverages, smoking cigarettes and snuffing were associated with decreased odds of high academic achievement.	Moderate

### Result compilation

A compilation of the study results based on statistical inference is presented in Table 3. A study was deemed positive if it had at least one statistically significant positive outcome measure, a study was deemed negative if it had at least one statistically significant negative outcome, and a study was deemed “no effect” if it showed no statistically significant results. A two-tailed *p*-value of 0.05 was considered statistically significant.

**Table 3:** Compilation of results from the studies

First author	Cognitive performance	Academic achievement	Morbidity risk factors	QoL/well-being
Hallstrom et al. (31)	N/A	N/A	+	N/A
Marlatt et al. (32)	N/A	N/A	+	N/A
Moschiano et al. (33)	N/A	N/A	+	N/A
Papoutsou et al. (29)	N/A	N/A	+	N/A
Sese et al. (34)	N/A	N/A	+	N/A
Smith et al. (30)	N/A	N/A	+	N/A
Walter (35)	N/A	N/A	+	N/A
Wennberg et al. (36)	N/A	N/A	+	N/A
Wennberg et al. (37)	N/A	N/A	+	N/A
Cooper et al. (50)	+	N/A	N/A	N/A
Defeyter and Russo (49)	+	N/A	N/A	N/A
Hjorth et al. (27)	+	N/A	N/A	N/A
Wesnes et al. (38)	+	N/A	N/A	N/A
Wesnes et al. (39)	+	N/A	N/A	N/A
Widenhorn-Müller (40)	+/−	N/A	N/A	N/A
Page et al. (41)	N/A	N/A	N/A	+
Richards and Smith (42)	N/A	N/A	N/A	+
Smith (51)	N/A	N/A	N/A	+
Boschloo et al. (43)	+	+	N/A	N/A
Burrows et al. (44)	N/A	+	N/A	N/A
Faught et al. (48)	N/A	+	N/A	N/A
Lien (45)	N/A	+	N/A	+
Littlecott et al. (5)	N/A	+	N/A	N/A
Ptomey et al. (28)	N/A	+	N/A	N/A
Sampasa-Kanyinga & Hamilton (47)	N/A	+	N/A	N/A
Stea and Torstveit (46)	N/A	+	N/A	N/A
Number of studies indicating positive effects	7 (100%)	8 (100%)	9 (100%)	4 (100%)
Number of studies indicating negative effects	1 (14%)	0 (0%)	0 (0%)	0 (0%)
Number of studies indicating no effects	0 (0%)	0 (0%)	0 (0%)	0 (0%)

+ = positive effect, − = negative effect, 0 = no effect, N/A= not applicable.

### Result of the search

**Fig. 1:**
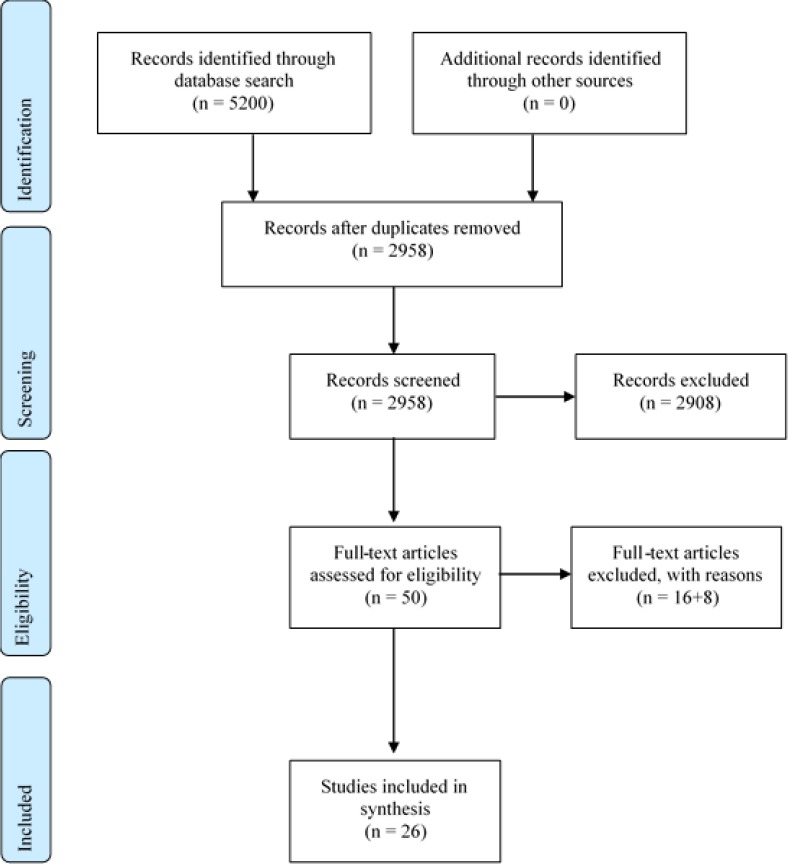
Flow chart of the work process: PRISMA 2009 Flow Diagram.

The flow chart presented in Fig. 1 illustrates the work process. Database searches identified 5,200 articles. After the removal of duplicates, 2,958 unique articles remained. Exclusion based on information given in title and abstract resulted in the removal of 2,908 articles. The full-text reading of the articles resulted in the additional exclusion of 16 articles that did not meet the inclusion criteria, and eight articles were excluded because of low quality (19–26) (see Appendix 1). Finally, 26 articles met the inclusion criteria and remained for further analysis.

## Results

The 26 studies included in the analysis are summarized in Table 2.

All studies were published between 2003 and 2017. The countries represented were the UK (seven studies); the United States (three studies); Canada, Norway, Australia, and Sweden (two studies each); and Denmark, Germany, Italy, and the Netherlands (one study each). Four studies were multinational. Nineteen of the included studies were observational. Four of these were also longitudinal with follow-up periods of 27 years in two studies, 21 years and 6 months in one study each. Five studies employed randomization. One study was a cluster-randomized controlled trial, three studies employed randomized crossover designs, and one study used a cluster-randomized crossover design. One study was a non-randomized controlled trial, and one study employed a separate-group design.

The number of participants included in the studies varied from 29 to 28,608. Based on the predetermined inclusion criteria, the age range of all children and adolescents included in the studies were from 6 to 18 years. Four of the studies had participants with a mean age below 10 years (27–30), the remaining 22 focused on studying participants with a mean age of 10 or more years (5, 31–51).

The studies included a variety of outcome measures and instruments. The outcome measures that met inclusion criteria were grouped into four categories. Nine studies comprise the category of morbidity risk factors associated with eating or not eating breakfast (29–37). Eight studies form the academic achievement category, with outcome measures that study links between breakfast and academic achievement (5, 28, 43–48). Six studies analyzed the association between breakfast eating and cognitive disorders, comprising the category of cognitive performance (27, 38–40, 49, 50). Finally, the quality of life and/or well-being category is made up of three studies, analyzing links between breakfast eating and QoL and/or well-being (41, 42, 51). No study concerning the cost-effectiveness of eating breakfast was found. Table 3 shows the compilation of the study results.

### Morbidity risk factors

All of the nine studies with morbidity risk factors as outcomes were observational studies. Five of the studies examined the association between breakfast consumption and the metabolic syndrome. Marlatt et al. found that higher levels of breakfast consumption were significantly associated with lower risk for insulin resistance as well as other risk factors for metabolic syndrome (32). This is in line with what both Sese et al. and Wennberg et al. concluded in their studies (34, 36, 37). The two studies conducted by Wennberg et al. were longitudinal with 27 years of follow-up. In both studies, they found an association between poor breakfast habits and future risk of developing metabolic syndrome. Hallstrom et al. studied the association between breakfast consumption and cardiovascular disease risk factors. Their results indicated that adolescents who regularly consumed breakfast had higher cardiorespiratory fitness and lower total adiposity (31). Significant effects of breakfast consumption on blood lipid levels, blood pressure, or insulin resistance were found in overweight/obese male participants, while no significant effects were found among female participants, regardless of weight status. Two studies examined the association between breakfast skipping and headache (33, 35). Both found that irregular breakfast eating is associated with headache.

### Cognitive performance

Various number of instruments and tests were used to measure cognitive performance among the seven studies included. All of them found that eating breakfast has positive effects on cognitive performance. Wesnes et al. concluded in both their studies that breakfast improved children’s attention (38, 39). Cooper et al. found that eating breakfast had particularly beneficial effects when study participants faced more cognitively demanding tasks (50). They also measured self-reported tension and calmness between the breakfast and no breakfast groups, but found no difference. In addition, they found that breakfast consumption improved response time measured with Sternberg paradigm, a memory-scanning task for short-term memory testing. This effect was not possible to confirm with other similar tests measuring response time. Wesnes et al. also showed that breakfast affects response speed variability. Participants who had breakfast showed lower response speed variability. Hjorth et al. and Widenhorn-Müller used the d2-test to measure selective and sustained attention and visual scanning speed. Hjorth et al. found a positive interaction between breakfast consumption and d2-test results (27). Widenhorn-Müller showed beneficial effects of breakfast on short-term memory and mood but not on sustained attention (40).

### Quality of life and well-being

The three included studies used different measurements to study the association between breakfast consumption and QoL and/or well-being. However, the results are consistent. Page et al. found that eating breakfast was one of the 12 significant predictors of self-rated health measures (41). Richards and Smith provided evidence showing that stress, anxiety, and depression were associated with skipping breakfast (42). Nevertheless, they highlighted the possibility that mental health might influence whether a person consumes breakfast or not. Smith et al. also found that breakfast consumption is linked to increased well-being (51).

### Academic achievement

All the eight studies that investigated breakfast consumption and academic achievement found positive associations between breakfast consumption and academic achievement. In a study conducted by Lien in Norway, the implications of skipping breakfast on mental distress and academic achievement were stronger for boys than girls and stronger for native Norwegians than immigrants (45). Burrows et al. concluded that dietary behavior overall was associated with higher academic achievement, but that breakfast was only significantly associated with the domain writing (44).

## Discussion

This review set out to find relevant outcome measures of eating breakfast on children and adolescents, for use in economic evaluations. The studies included focused mainly on studying the effects of eating versus not eating breakfast, and they were divided into four categories of study outcomes, namely morbidity risk factors, cognitive performance, quality of life and well-being, and academic achievement. All studies included in the analysis met the inclusion criteria of this review and were judged to be of at least moderate quality. Consequently, the excluded studies either failed to meet the inclusion criteria or had a low study quality rating.

There was a notable variation in the study design. In general, studies were either of observational or experimental design. Even though, randomized control trial is the only study design for drawing causal relationship, the observational study still is important to identify associations. Whether children eat breakfast or not and whether it has any substantial effect on study outcomes might very well be derived from a large number of unobserved influential factors, such as the children’s social environment and parental support. Thus, assigning the effects of eating breakfast to its sole nutritional value may be problematic.

The review showed associations between eating breakfast and cognitive performance, academic achievement, quality of life, well-being, and morbidity risk factors. When results are consistently positive, different forms of bias need to be discussed. There is always a risk of publication bias that could contaminate this type of review, which could have overestimated the strength of the evidence (52). In addition, reformulations of initial study hypothesis to better suit data might affect results. This is known as HARKing—hypothesizing after the results are known (53). Also, the lack of control for confounders may have affected the results. As it is not possible to adjust for all confounders, it is difficult to establish a clear causal link between intervention and outcome (54). Studies that examine the association between breakfast and weight/obesity have been thoroughly studied and reviewed before. Therefore, such studies were excluded from this review. However, despite the exclusion of the weight/obesity effects of eating breakfast in this review, it should be noted that it is relevant to include weight/obesity in the economic evaluations of breakfast.

To only include studies performed entirely or partly in countries listed by the CIA as advanced economies, except Japan and Taiwan, restricts the relevance of results to these countries. The potential exclusion of relevant studies from other nations is thus one limitation. In the assessment of study quality, the risk of incorrect classification is contingent. Although, by proactively disregarding the study results in the articles included when performing quality assessment, the authors took measures to limit the risk of study quality misclassification.

Our interest in finding and valuing effects that can be attributed to breakfast eating emanates from the question of the cost-effectiveness of breakfast interventions. Our study cannot answer that question, but shows that breakfast eating is associated with effects that could be used in economic evaluations, especially when using decision analytic modeling, calculating the long-term cost and effects of intervention. For instance, improved cognitive performance and academic achievements may influence long-term effects on an individual’s productivity through improved school results, grades, and higher education. Further, this gives higher human capital, which can be reflected in higher income, both for the individual and for society. Improvements in quality of life and well-being will have an immediate effect on the QALY weight of a child or adolescent but also, if the effects are sustained, on long-term QALY gain. Finally, the health effects mediated by a reduction of long-term morbidity risk factors, such as obesity, will contribute to lower morbidity/mortality and a reduction in healthcare costs. In order to answer the questions regarding the cost-effectiveness of breakfast and interventions promoting breakfast eating, we need to know more about how the identified effects influence the societal cost and the individual’s QALY gain. There is also a need to develop models that can simulate breakfast and breakfast interventions’ long-term costs and effects. In this study, we have started that work by identifying important model parameters.

## Conclusions

The overall assessment of the studies indicated positive associations between eating breakfast and study outcomes that measure cognitive performance, academic achievement, quality of life and well-being, and morbidity risk factors in high-income nations. How these outcomes influence societal costs and individuals’ QALYs require further research.

## Conflict of interest and funding

The present study was sponsored by a grant from Arla Food AB. The sponsor of the study had no role in study design, data collection, data analysis, data interpretation, writing of the report, or the decision to submit for publication. The authors had full access to all the data in the study and had final responsibility for the decision to submit for publication. The authors declare no conflicts of interest.

## Appendix 1

Studies that were excluded because of low quality and reasons for exclusion are presented in Table A1.

**Table A1:** Excluded studies because of low quality, with reasons for exclusion

Exclusion no.	First author (year)	Title	Reason for low-quality rating
1	Adolphus et al. (2015) (19)	The relationship between habitual breakfast consumption frequency and academic performance in British adolescents	1, 6
2	Benton and Jarvis (2007) (20)	The role of breakfast and a mid-morning snack on the ability of children to concentrate at school	1, 3, 5
3	Karatzi et al. (2014) (21)	Dietary patterns and breakfast consumption in relation to insulin resistance in children: The healthy growth study	1, 7
4	Kral et al. (2012) (22)	Effects on cognitive performance of eating compared with omitting breakfast in elementary schoolchildren	1, 3, 5
5	López-Sobaler et al. (2003) (23)	Relationship between habitual breakfast and intellectual performance (logical reasoning) in well-nourished schoolchildren of Madrid (Spain)	2
6	Maffeis et al. (2012) (24)	Breakfast skipping in prepubertal obese children: Hormonal, metabolic and cognitive consequences	5
7	McIsaac et al. (2015) (25)	The association between health behaviors and academic performance in Canadian elementary school students: A cross-sectional study	1, 6
8	Overby et al. (2013) (26)	Self-reported learning difficulties and dietary intake in Norwegian adolescents	1, 3

Matters causing low-quality rating: (1) No RCT, (2) lack of adequate control group(s), (3) lack of control for confounders, (4) insufficiently described experimental design, (5) insufficient statistical power, (6) non-relevant outcome measures, and (7) non-consistency between reported results and conclusions.
